# Exploring the integration of IoT systems in interior design and the built environment: A systematic review

**DOI:** 10.1016/j.heliyon.2023.e22869

**Published:** 2023-11-25

**Authors:** Yaman Sokienah

**Affiliations:** Yarmouk University, Department of Design and Applied Arts, Irbid, Jordan

**Keywords:** Internet of things, Interior design, Built environment, Smart homes, Technology

## Abstract

This research aims to advance the understanding of the integration of Internet of Things (IoT) systems in interior design and the built environment. To assess the current state of research in this field, a systematic review method was used. Multiple databases were searched using keywords such as "smart homes," "smart houses," "IoT and interior design," "IoT," and "the built environment," yielding 475 articles. Following the application of inclusion criteria, 14 articles were chosen for analysis. The findings of this review indicate that, while IoT system integration in the built environment is still in its preliminary stages, it has significant potential for global development in the future. By investigating current and future approaches to IoT systems in interior design and the built environment, the paper adds to the body of knowledge.

## 1 Introduction

The advancement of technology has provided the world with the ability to connect devices through the Internet of Things (IoT). The advancement of technology has provided the world with the ability to connect devices through the Internet of Things (IoT). This technology has been integrated into many sectors, including the built environment. The Internet has changed and continues to change people's lives by allowing connectivity anytime with anyone who has Internet access [1]. In addition, the availability of cheap, low-cost Internet transmitters, sensors, and routers allows most people to install them in their homes without breaking the bank [2].

As the number of Internet-connected devices increases, a study showed that by 2020, there will be more than 50 billion electronic devices connected to the Internet, with a prediction of this number reaching over 200 billion [3]. This trend of connecting our devices leads to new concepts and functions for regular users beyond daily Internet use. For example, people are using the Internet to control their homes and the spaces they use, and one of these functions is enabling the IoT [4,5].

IoT is a concept that allows users to connect everyday objects and smart devices to the Internet, such as home appliances and mobile devices. When these objects and devices are connected to the Internet through a network and infrastructure, it can be referred to as an IoT system (Gaikwad et al., When these objects and devices are connected to the Internet through a network, and Internet infrastructure can be referred to as an IoT system [1,6]. The idea of connecting these objects and devices to the Internet is that they can communicate and configure themselves without human intervention [1].

Improvements on the Internet of Things and Big Data Technology and Their Potential Impact on Many Areas of Human Life [[7], [8], [9]]. Implementing IoT systems in the built environment reduces energy consumption and lowers energy costs ([10]. Additionally, it is being implemented in healthcare systems where it can lower healthcare costs through personalized care and healtsh insurance systems where the patient can be monitored remotely, reducing doctor visits [7,10].

It can be observed that many architects and interior designers are shifting their focus to non-traditional design concepts and moving beyond stereotypical homes and living spaces [11] It has also been noted that smart spaces can be considered sustainable and green buildings because IoT systems can reduce energy, water consumption, and increase comfort within interior spaces [12].

Moreover, researchers worldwide are developing and studying IOT systems to benefit people using those devices daily, such as in their homes and interior environments [13]. Therefore, this paper aims to contribute to the body of knowledge regarding IoT systems and their implementations in interior design and the built environment. It also explores the available and future approaches to IoT systems and their relationship with the interior design industry.

As technology advancements are rapidly developing in the past decade, one of the significant fields of technology that grabbed the attention of researchers from multiple fields is the Internet of Things "IoT" [[14], [15], [16]]. It can be hard to find a definitive answer on defining IoT in the literature. However, on a fundamental level, and not to get too technical in this paper, IoT is where one can connect daily objects to the Internet through receivers' interpreters and networking capabilities [17,18]. Those objects with these capabilities can communicate with other devices to distribute or receive specific information to function in a specific way [19,20]. However, IoT can be more complex than previously stated in terms of connecting our world of objects with a virtual world of information technology to have more connections and services [10].

As an emerging technology, many people have different perceptions of IoT. However, most of the IoT systems being used or expected to be used in the built environment are sensor-based technology. The primary function of these IoT systems is to collect data from sensors within the space and transmit the collected data to a larger network through Bluetooth or Wi-Fi for processing and decision-making. Their cost and energy efficiency make sensor-based systems more popular [7] Therefore, it can be concluded that, IoT systems provide the built environment in which they are installed with the ability to collect data, transfer collected data, act and react based on collected data that matches a specific algorithm, and store and analyze collected data [7,21].

IoT technology has many functions that improve the quality of life for occupants when implemented within interior spaces. These functions are related to lighting control, thermal comfort, life support assistance, energy management, and HVAC systems control [10,22]. However, there are few integrations of IoT technology within interior spaces regarding furniture and how using such technology can change the layout and planning of interior spaces [23]. Considering this, it is important to investigate the feasibility of incorporating IoT technology into the interior design process when planning spaces and specifying their furniture to be used in them. This paper provides a baseline for interior designers, who may not have extensive experience with IoT technology, to integrate it into the built environments they design.

After checkig the published research, it was clear the there is a lack or review studies that focuses explicitly on IoT systems and its implication for interior design and the built environment from a comprehensive perspective. Since there is a fast pace of advancement in the technological development in IoT in its nature, new insights can be gained from an updated review [1].

The rapid development of IoT systems and technology as a field requires a reguiler review to keep the research audience up to date with the latest development. This paper provide a comprihansive and updated review of these trends, as a it adds to the existing body of knowledge.

Hence, this review aims to offer valuable insights for interior designers seeking to navigate the emerging landscape of IoT-enhanced design by addressing this lesser-studied aspect of IoT integration. This systematic review seeks to advance the understanding of IoT systems' effective and informed integration within interior design and the built environment. Given the rapid development of IoT technology and its implications for interior design, there is a clear and urgent need for this focused review.

### 1.1 Study aims

The Internet of Things (IoT) is revolutionizing numerous sectors through its ability to connect devices and create 'smart' environments [1,2].It is particularly transformative in the built environment, where it holds the potential to enhance the functionality and sustainability of interior spaces [2]. This paper presents a systematic review of the integration of IoT systems within interior design and the built environment, an area which despite growing interest, remains underexplored in the literature.

As the pervasiveness of IoT continues to rise, it is predicted that by 2025, the number of Internet-connected devices could surpass 200 billion [3]. This dramatic growth propels a significant shift in how individuals interact with their surroundings, as IoT allows control over homes and spaces with greater efficiency and convenience [5]. Despite its promise, the implications of this technology for the design of interior spaces have yet to be fully articulated, providing a strong impetus for this systematic review.

The adoption of IoT is already manifesting in several sectors, driving advances in energy efficiency, healthcare, and the design of living spaces (Koo et al., 2016; de Boer et al., 2019). It is also triggering a change in basic assumptions among architects and interior designers, who are beginning to embrace innovative design concepts and approaches enabled by IoT technology (Desjardins et al., 2019). However, the potential applications and impact of IoT within the interior design realm remain under-studied, creating a significant research gap that this review seeks to address.

This systematic review, therefore, aims to advance understanding of IoT's role and potential in interior design and the built environment, drawing on global research into the development and application of IoT systems. Recognizing the rapid pace of technological progress in the IoT field (Khan & Alam, 2021; Shi et al., 2019; Zhan et al., 2014), this review also considers how IoT systems, primarily sensor-based, are deployed within built environments to gather, transmit, and respond to data, and how these capabilities may influence future design strategies (Koo et al., 2016; Rodrigues et al., 2018).

Finally, the review acknowledges that while the benefits of IoT in terms of lighting control, thermal comfort, energy management, and HVAC system control have been well-documented, there is scant literature on the integration of IoT in furniture and space planning within the interior design process (Frischer et al., 2020). As such, this review will explore this lesser-studied aspect of IoT integration, offering valuable insights for interior designers seeking to navigate the emerging landscape of IoT-enhanced design.

By shedding light on the current state of research in this area and identifying areas for future exploration, this systematic review aims to contribute to the effective and informed integration of IoT systems within interior design and the built environment.

## 2 Methods

This paper uses the systematic review research method. To do this, several keywords were searched in multiple databases. The keywords used were: "Internet of Things"; "IoT"; "Smart Home"; "Smart Space"; "Interior Design"; "IoT Security"; "IoT Privacy"; "IoT in Healthcare"; "IoT in Residential Spaces"; "IoT in Commercial Spaces"; "IoT System Limitations"; "IoT System Implementation. " These keywords were used in various combinations to retrieve the most relevant articles for the review. Also, these keywords were combined with the terms "OR" and "AND" followed by "Internet of Things" or "IoT." The keywords were used in searches on the following databases: Google Scholar, Scopus, IEEE Xplore, and ScienceDirect. These databases cover many papers on IoT applications in smart homes from an interdisciplinary perspective.

## 3 Exclusion criteria

In our systematic review, we implemented a two-step exclusion criterion to streamline the collected literature and focus only on the most relevant papers to our research. Our exclusion criteria were:

1 Articles must discuss aspects of the 'smart home' concept in relation to IoT and offer new insights or contributions to the field. Literature that merely mentioned the term 'smart home' or IoT without a substantive discussion or contribution were excluded.
2 The selected articles must provide a detailed examination of the IoT-based smart homes and their underlying communication mechanisms. Studies that merely used the term 'IoT-based smart homes' without delving into the technicalities and particulars of the IoT communication systems were also excluded.

**Fig. 1:**
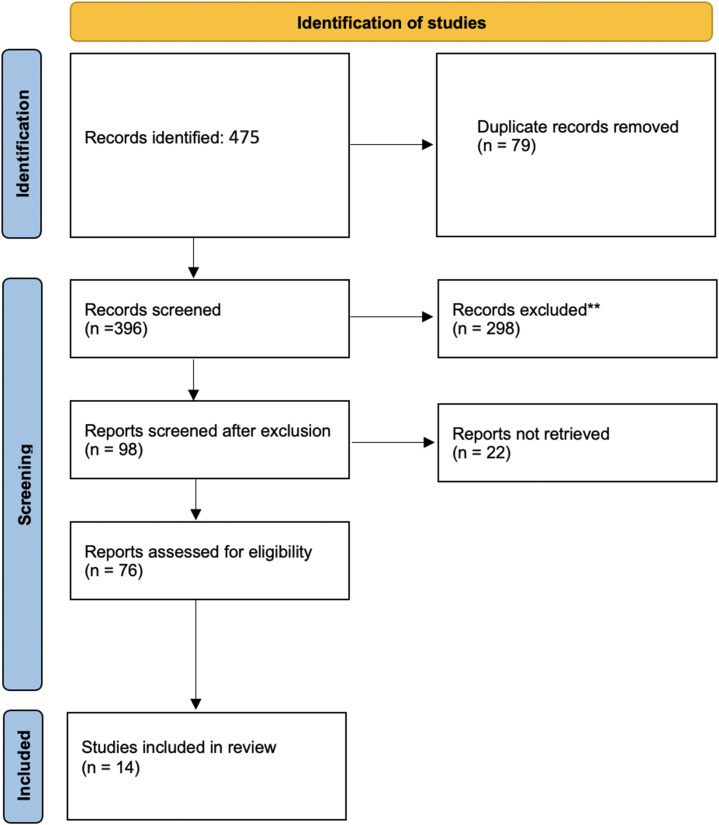
PRISMA Protocol Paper search flow chart.

It is also important to note that our search was restricted to papers published in English due to language capabilities. In this way, we assessed 475 articles related to the topics under investigation. Given the lack of a universal building code or guideline on integrating IoT systems within the built environment, our selection was based on the quality of the subject covered in the paper and its theoretical considerations of the topics (Fig. 1). We believe that this clear and focused approach helped maintain the integrity and relevance of our research. In the PRISAM (Preferred Reporting Items for Systematic Reviews and Meta-Analyses) flow chart you can see the process of exclusion along with the number of studies used in the review.

Outcome measures will include general and specific quality-of-life improvement devices and strategies that can or have been implemented within the built environment. Security and energy efficiency are particularly relevant to users and the reasons for implementing IoT systems in the interior space of the built environment. Furthermore, specific outcomes relevant to smart furniture will be grouped into categories and subheadings, such as smart desks for remote work and safety within the built environment in both residential and healthcare spaces [[24], [25], [26], [27], [28]]. Depending on the included studies, these categories and groups may be modified, as necessary. The risk of bias is kept to a minimum in this paper because there are no trials or direct interventions with the built environment users or occupants.

Review data were processed, and a narrative summary was written, focusing on how the findings related to the study's stated goals. Data analysis involved summarizing the significant benefits of using IoT systems within the built environment and conducting a thematic analysis of the included papers to interpret the emerging themes and patterns. Finally, a table of captured critical categories and a summary of key findings and their interpretation regarding the integration and implementation of IoT systems, both positive and negative, within the built environment were provided.

### 3.1 Study characteristics

475 papers were found and collected from the search. These papers were from the following databases: IEEE Xplore, ScienceDirect, Scopus, and Google Scholar. They were all published research and articles. In the first round of filtering round, duplicate papers were removed, leaving 396 papers. Then, in the second round, the title and abstract of each paper title and abstract were reviewed and scanned, resulting in the exclusion of 382 papers due to their irrelevance. 14 papers met the inclusion criteria that directly connected the built environment and IoT systems from different perspectives. The 14 papers that met the inclusion criteria are summarized in Table 1.

**Table 1:** Studies included in the systemic review.

Author, year	Aims	Study design	Main findings
[29,30]	It created a "Smart Kitchen Cabinet" that can identify what is in the pantry.	Experimental	The system automatically prepares a shopping list when an item exceeds a certain threshold based on family members' needs and consumption patterns.
[30]	addressing the issue of the unsanitary state of public restrooms	Experimental	
[10]	This study aims to determine whether and how Internet-of-Things (IoT) skills affect the adoption and (planned) use of IoT technology.	online survey	People's evaluations of their IoT abilities, the importance of pragmatic reasons for its acceptability, and the fact that IoT proficiency needs Internet proficiency.
[31]	This article looks at the sensor technologies used by smart homes, specifically concentrating on perceiving the immediate surroundings and sense mediated by the underlying infrastructure.	Review	Users, their loved ones, and caregivers, as well as their doctors and therapists, may all find something in the latest sensor technologies for smart homes that suit their needs.
[32]	Provides details on developing a cheap smart door sensor that can be used to monitor entry, exit points, and alert the user through an Android app.	Experimental	With the help of low-cost components like Elegoo and Raspberry Pi microcontrollers and R.F. signals for inter-device communication, a home user may monitor the opening and closing of a specific door through the Internet of Things.
[33]	This research has resulted in the development of a smart kitchen fire prevention system that includes the following features and devices.	Experimental	The suggested system features flame, temperature, and gas detection and automated door unlocking, allowing the community management team and emergency professionals fast access to the home to battle the fire.
[34,35]	Explain how the Internet of Things (IoT) devices are enhancing health service delivery and how IoT will revolutionize and disrupt health care throughout the world over the next decade.	Review	IoT-based health care can boost system efficiency and improve population health.
[36]	Build a smart home security system that can be monitored remotely from anywhere.	Experimental	An "emergency email" and text message are sent to the user, who may then see a live video feed of their house on their smartphone through Wi-Fi or LTE.
[37]	Remote surgery, computer-assisted surgery, and remote patient monitoring and care have reached their apex due to advancements in information and communication technology and artificial intelligence (A.I.) capabilities on the Internet of things.	Review	IoT devices provide an effective, non-invasive, non-destructive, low-cost, accurate, and time-saving option for real-time monitoring of the user's health.
[38]	Keep our surroundings neat. This document contributes to the prevention of disease spread via communal toilets.	Review	Using a hardware-based prototype module with all automated sensors and new Internet of things technology, we will increase public awareness of proper hygiene and adopt more intelligent technologies.
[32,39]	Complements previous research on the causes of latrine construction, adoption, and continued usage throughout time.	ethnographic and technical methods	This study adds to the fields of sustainable development and global public health via the establishment of a sanitation theory and framework.
[40]	Concentrates on ammonia gas sensors and sensor systems.	survey	Often used in medical applications like breath analysis equipment, these systems lend themselves well to downsizing and integration, making them useful for monitoring tiny gas quantities.
[12]	How to manage and control the increasing number of diverse home appliances efficiently and conveniently to achieve a more comfortable, secure, and healthy living environment has been proposed; the solution is a smart control system based on the technologies of the Internet of things. This solution has been proposed as an answer to the problem.	Experimental	Adding a new appliance to the control system or removing an item is very straightforward. The intelligent control system can monitor, regulate, and manage appliances and provide home security, energy statistics, and analysis. It also manages and analyzes energy use.
[13]	By providing a unified taxonomy and describing the characteristics and features of emerging studies on this problem, the authors highlight new, disruptive technologies and provide terrain for future research. Elucidate new, disruptive technologies and establish a research landscape.	Review	The research highlights a few omissions in using the communication functions of smart home devices. Consequently, several management and regulatory recommendations for these components have been offered. To reduce their power consumption, maintain a safe and secure environment, accomplish accurate and dependable management of a range of control devices, and increase the quality of their user experience, users must carefully follow the offered instructions on how to utilize these components.

## 4 Results and discussion

### 4.1 Brief exploration of the IoT system components

Many built environment practitioners may have a basic understanding of IoT systems but may not be knowledgeable about the complex, intricate details involved, from hardware devices to software and programming issues. Therefore, an introduction to the components of an IoT system is necessary for this paper.

An IoT system is made up of two major components. Hardware and software components are set up and connected to the Internet, along with several types of sensors based on what the user wants to measure [41,42].

An IoT system is made up of two main components: hardware and software. These components are set up and connected to the Internet, along with several types of sensors based on what the user wants to measure [41,42].

The user interface allows the information provided by the end-user to be actionable. For example, a user warning might be issued through an email, text, or notification [3]. For instance, a text message alert could be sent when the temperature in aa company's cold storage temperature rises above a certain threshold. An interface is also provided that routinely polls the system to check its status [43].

### 4.2 IoT systems in healthcare setting

Findings revealed several existing strategies for implementing IoT systems within the built environment that are designed and implemented to improve the occupants' quality of life of occupants and reduce energy consumption. Many of these strategies, when implemented in healthcare spaces, are aimed at improving the lives of patients and reducing the risk of falls and detecting such, many strategies improve patients' lives, reduce the risk of falls, and detect events [34]. However, healthcare studies implementing IoT strategies are often trial experiments and are not supported by a solid evidence-based theoretical aspect [34,44].

Other studies have developed, designed, and tested innovative approaches for using IoT systems in smart homes to control water consumption and temperature. However, most of these studies were very technical and may be beyond the reach of interior designers and architects without deep understanding and experience with IoT systems. While all the studies stressed the importance of IoT systems and demonstrated a clear potential for the benefits of IoT system implementation within the built environment to improve quality of life, other studies emphasized the need for further experimentation before IoT systems can be fully implemented in the interior spaces of the built environment [35].

Most of the studies that implemented IoT technology within a healthcare environment had a significant focus on using cameras and sensors to provide healthcare services to elderly people, where privacy was a significant concern, especially when using cameras to detect facial expressions to evaluate and understand the emotional state of the patient in order to act on those emotions [45]. In addition to privacy issues with the implementation of IoT systems in healthcare spaces, these systems have the potential to predict emergencies, particularly for elderly people with special needs who live alone or in a minimal care facility [45]. To address privacy issues, it is proposed to encrypt the collected information using fog computing technology and store it at the network edge rather than using cloud-based storage systems [46]. To address privacy issues, it is proposed to encrypt the collected information using fog computing technology and to store it at the network edge rather than using cloud-based storage systems [46].

The findings of this review suggest that implementing IoT systems within healthcare spaces can positively impact emotions and can change the health of the person using the space or being monitored [47]. The findings of this review suggest that implementing IoT systems within healthcare spaces can have a positive impact on emotions and can change the health of the person using the space or being monitored [47]. This can be beneficial in taking quicker action if a situation arises at any time of day. In contrast to traditional healthcare facilities, where there are no automatic situational detection systems, actions are correlated with human visits and scheduled check-up times implemented by medical staff [34]. This can reduce stress on staff as they have a reduced number of visits per day or night. However, IoT patient monitoring systems are still developing and improving the quality of situation detection and privacy [48], IoT patient monitoring systems are still in development and are improving the quality of situation detection and privacy [48].

IoT systems can alert healthcare providers about emergencies and provide monitoring capabilities for at-risk patients with different symptoms [46,47]. However, as it has been found from the search, this implementation of the IoT system is still in its infancy and is undergoing constant modification and development. Integrating IoT systems within the built environment can lead to more complex functions and usage. However, little research has been done on IoT systems in healthcare spaces. As the number of people with disabilities increases and the number of older adults ages in place, smart home technology may offer a solution to alleviate the burdensome caregiving responsibilities of family caregivers and healthcare providers and promote independent living [31].

### 4.3 IoT systems and energy efficiency

Energy conservation is a significant factor that must be considered when constructing or occupying any space. One way to conserve energy in smart spaces is to monitor energy consumption within a given space, analyze the collected data, and improve energy consumption [49].

A cloud based IoT network can help monitor energy conservation in smart homes [50]. In a smart home, numerous IoT gadgets are deployed, and data is monitored and transferred to the cloud via IoT communication devices [50]. In a smart home, numerous IoT gadgets are deployed, and data is monitored and transferred to the cloud via IoT communication devices [50,51]. These gadgets act as a gateway in the house, transmitting data collected by monitoring systems to an online cloud service [50,51].

The use of renewable energy in buildings is becoming more common. According to Ref. [52], solar energy is the largest contributor to domestic energy consumption, while wind, biomass, and geothermal energy contribute less and are more constrained due to urban topography and climatic conditions [53]. Using renewable energy also shows significant savings from transmission energy losses and may provide traditional primary energy savings [52].

Grid and smart home energy management and design were tested, and the results were used to develop an energy timing interface model [54]. The results of the tested model showed the accurate calculation of energy consumption and supply, and it helped provide effective energy management, optimization, and load scheduling on a city scale. These models can significantly improve energy consumption when implemented on a large scale, not just a few houses or structures in a city [54].

The smart home control system demonstrated a significant impact on energy conservation when using IoT systems within a smart home. It was found that using just a few sensors saved about 12 % of total energy consumption within space [55]. It can also be concluded that if the systems had more sensors and tuning, there would be an even greater amount of energy conservation.

In conclusion, the advancement of technology has provided the world with the Internet of Things (IoT), which has been integrated into most sectors around the world worldwide, including the built environment. By 2020, it is estimated that there will be over 50 billion Internet-connected devices, with a prediction of this number reaching over 200 billion by the year 2021. The main function of IoT systems is to collect data from sensors within a space and transmit this data to a larger network for processing and decision-making. Implementing IoT systems in the built environment can improve the quality of life for occupants, reduce energy consumption and costs, and lower healthcare costs with personalized care. However, privacy concerns must be addressed, particularly in the healthcare sector, and they need to be addressed. Energy conservation is a significant factor that can be improved with the use of IoT systems, and adopting these systems can help users monitor and determine their energy consumption rates, allowing them to make energy-efficient choices [[56], [57], [58]]. However, more development is needed before these systems can be widely used by the general population can widely use these systems [59].

### 4.4 Kitchen automation system

People conduct multiple tasks in their daily lives, and the kitchen is one of the significant spaces that people use daily; with so many activities going on in the kitchen, the temperature will change quickly [33]. In addition to temperature, using gas stoves offers a significant fire risk. Based on this, the use of IoT in the kitchen is essential to keep the kitchen thermal comfort constantly comfortable and to avoid the dangers connected with the use of gas burners [60]. Furthermore, People who have lost their autonomy due to a cognitive disability typically have to do necessary daily tasks (like cooking) using tools and equipment designed for healthy persons, which do not account for their cognitive impairment [33].

Also [33], have developed and tested an IoT system that detects a gas leak, flame, or excessive temperature in the kitchen. The systems could trigger an alarm warning to the users and the authorities and shut off the gas supply. Also, the system is connected to the house's main entrance, which makes it unlock for people to escape the space and allows first responders to enter the space in case of a fire. This can significantly minimize the number of fatalities in kitchen fires each year [33,36,60,61].

Previous research has planned, built, and tested utilizing an IoT system with multiregional sensors within the kitchen space. The testing results showed that an IoT system could deliver SMS notifications anytime there is a high gas concentration in the air. As a result, this system may be used in private houses and public establishments like hotels and restaurants. In addition to being fully automated, modern kitchens also have safety features like gas leak detectors [61].

IoT systems can also be implemented in kitchens by installing sensors and identification devices within the cabinet space. Sensors and identification devices in cabinet spaces can help grocery-level management by measuring product weight before and after use [62]. Then an automated shopping list is generated and updated based on the weight readings, where the system notifies the user about the lack of a specific product that is running low. Also, this system can track the number of specific products you buy in a specific period [29]. This can help each household adjust its shopping list and help with monthly budgeting.

### 4.5 Bathroom automation system

It is well known that poor sanitation and hygiene of bathrooms can negatively impact users' health and welfare [39]. Bathrooms are spaces either in residential, commercial, or healthcare facilities where they can be a space to contract germs and microbes ([39]. Therefore, automating bathrooms came to provide and maintain a cleaner, more hygienic, healthier bathroom space [37].

The parts of IoT systems in the bathroom have a variety of sensors starting from sensing the presence of the user to automating flush systems and checking natural ventilation within the space [38,47]. The user presence sensor is activated when a person enters the bathroom. This sensor can activate lighting and power outlets if available. Also, IoT systems help identify whether the bathroom is clean; if the values exceed the pre-determined threshold, then an alert is sent to the cleaning team in the public space [30,38].

After finishing using the bathroom, a flush system is automatically activated [63,64]. This can be used in a publish or a private toilet where it can reduce water consumption and make using the bathroom more accessible [65]. During usage time, sensors keep checking ammonia and odor levels in the air; if those levels are above the pre-determined threshold, a space freshening system is activated until the level of ammonia and odor are in the acceptable range [38,40,66].

Furthermore, if a person is trying to use a public bathroom, IoT systems can check the availability of the bathroom. Also, this can provide the person with an available bathroom in case the bathroom on that specific floor is fully occupied. This reduces waiting times in large buildings and populated events for public bathrooms. Also, the IoT system can notify security if someone is taking too long in the bathroom to alert them that a user might be facing a health emergency [67,68].

One can notice the vast benefits and potential of having an IoT system installed within the bathroom's interior spaces, whether in a residential or commercial space. Those benefits can be more developed to reach an ideal efficiency and ease of use level for the public.

### 4.6 IoT systems for security, safety, and privacy in interior space

As one digs deeper into the field of IoT. The system, functions, and uses become more important for home and space users. Security, safety, and privacy are some factors that can be critical and hugely beneficial from IoT technology. As for home security, drastic developments have been made through IoT systems, such as switching from only an on/off alarm system to an intelligent space security IoT system where it collects data through sensors distributed within the space and sends the collected information to the users' phone and the authorities through the Internet regardless of the Internet action while monitoring the situation via live feed at the same time [36,69]. Therefore, having an IoT security system will assist users in protecting their houses and space by mounting the system on their doors or windows and monitoring activities via their smartphones [32].

Furthermore, as part of using IoT systems for security. As mentioned previously, it can also be used to monitor the space for potential safety issues such as room temperature and humidity levels, the flame on the stove, rain conditions, and the presence of intruders. They also control the air conditioning system and detect if a person has filled, or he/she is in distress [32,36,70].

The advantages of using an IoT security system within the space come from several factors, such as the low cost where a user can install an IoT security system, its affordability, and the minimum efforts needed to maintain the system [[70], [71], [72]]. Also, an IoT security system allows the user to monitor the situation and decide whether to notify the authorities or check if it is a false alarm, which can benefit first responders ([[73], [74], [75]].

Due to the previously mentioned advantages, it can be concluded that ease of use and affordability can be significant factors in making IoT security systems trending within interior spaces, whether in residential or commercial spaces. Also, ease of installation is one of the factors that can be included as part of the advantages of IoT systems, where it does not require significant changes to the infrastructure of the space, and many of the devices used for the system are wirelessly connected.

### 4.7 IoT System Limitations

As systems are being used increasingly within interior spaces and are now considered part of the design process more than ever, specialists try to warn people about some downfalls and limitations of IoT systems that anyone who uses them should be aware of. Furthermore, it is argued that manufacturers are focusing more on the energy consumption of each device and sensor without digging deep into solving some privacy and security issues that can come from using an IoT security system in the interior space [36].

In general, IoT-based security systems have concerns since they rely on a wide variety of Internet-connected devices, each of which has its own unique set of privacy and security risks [3,31]. More research into security and privacy concerns is needed to develop solutions that will keep users of smart homes safe from harm, both in the physical and digital realms.

Another limitation of using IoT systems in the interior space is that it requires some understanding and skill with using smartphones and technology. However, not all the population has these skills. This issue can be overcome with the proper training and development of a simple and easy-to-read guidebook and guideline fitted for each installed system that gives clear and direct instructions to the users [10].

Table 2 summarizes the key findings derived from the literature.

**Table 2:** A comprehensive summary of key findings.

	Key finding	Rational
*1.*	IoT systems and Energy Efficiency	IoT devices can monitor and manage energy usage in real-time, enabling significant energy conservation. This finding is supported by the research of [12] highlighting an intelligent control system for home appliances based on IoT technologies, optimizing energy usage.
*2.*	Kitchen Automation System	IoT systems can enhance kitchen safety by detecting hazards such as gas leaks and elevated temperatures. This is supported by the works of [29,33] who implemented IoT technologies in kitchens, like the "Smart Kitchen Cabinet" and a fire prevention system, respectively.
*3.*	Bathroom Automation System	The introduction of IoT systems into bathrooms can improve hygiene and user experience by automating certain functions [38]. work on hygiene in communal toilets supports this, showing that IoT can increase public awareness of proper hygiene and promote the adoption of smarter technologies.
*4.*	IoT Systems for Security, Safety, and Privacy	IoT systems significantly enhance home security and safety, offering real-time monitoring and alerting capabilities. This is backed up by Ref. [32] development of a cheap smart door sensor, and [36] creation of a smart home security system that can be monitored remotely.
*5.*	IoT Systems in Healthcare	IoT devices also find significant use in health service delivery and remote patient monitoring. This is supported by Refs. [34,37] work, highlighting the role of IoT in revolutionizing health care and enabling non-invasive, accurate, and time-saving real-time monitoring.
*6.*	IoT System Limitations	Despite the advantages, IoT systems have potential drawbacks including privacy and security concerns and require a certain level of technological understanding from the users. This is highlighted by Ref. [10] study on IoT skills affecting the adoption of IoT technology, and [13] taxonomy of emerging studies on this issue, underlining the need for careful management of IoT components for safety, security, and power consumption considerations.

## 5 Conclusions

This paper aimed to systematically evaluate and assess the IoT systems' role in interior design and in creating smart homes and spaces. Several studies have defined IoT systems and their importance to smart homes, along with providing information on creating smart homes through implementing sensors and collecting data. It can be said that there is an urgent need to study the field of smart homes since the topic is trending, and people are getting more interested in smart homes and spaces.

Systematic reviews on Interior Design and built environment and their relationship with IOT studies are incredibly important to guide the implementation of IoT tools to provide an overview of life and space improvement through the right integration between design and technology.

The role of the interior designer in this field is expanding, and more information regarding smart home and spaces are needed for interior designer to convince and provide their clients with the best services they can. However, it can be noticed that most of the published studies are very technical, and it can be challenging for an interior designer to understand the smart home's components thoroughly. Therefore, a collaboration between architects, interior designers, and I.T. Specialists should be undergoing in the industry of smart homes and spaces.

The benefits of IoT systems were identified in various parts of space. In healthcare facilities, it can help monitor patients in their homes or at the care facility and alert healthcare providers at any point of the day. Therefore, this can reduce trips to healthcare facilities and the workload on healthcare providers. Also, the benefits of IoT systems were identified in terms of residential spaces based on each space where it is implemented. Furthermore, most of the research focuses on the advantages of IoT systems in space surveillance, where spaces can be monitored throughout the day, and the users can be notified about any predefined issue. This can correlate with safety and security within space. The tole of IoT systems is expanding in the practice of interior design, more research improves its benefits for users of the space, and healthcare providers in healthcare facilities can help meet the ever-changing demand for better spaces and healthcare facilities.

This systematic review is a starting point for further research regarding smart homes and space implementation. This review revealed that the integration of IoT systems within the built environment is still under development. However, this field has immense potential to expand on a global scale, and the estimated market value of such an industry to be around 98 billion USD by 2025, with the number of connected devices to the Internet estimated to be six times the world population [76]. Smart homes and spaces have a promising future, with immense potential still to be fulfilled [22]. These results provide credence to the argument that smart home space needs a design-specific strategy to adequately answer major obstacles in the design industry, such as delivering safe, secure, and low-energy consumption places.

Studies show that to transform conventional indoor environments into "smart," multi-level, multi-strategy system techniques must be used. Those upgrades would be highly effective for healthcare providers and space users. Also, embedding those upgrades and interventions within the interior space would provide considerable security and energy savings. However, more high-quality research is needed to confirm energy-saving claims. Hopefully, this analysis's findings will encourage more studies and actual IoT practitioners and interior design professionals to work together more closely to produce integrations of IoT systems and the interior environment.

Future systematic reviews regarding IoT systems and interior design would benefit from reporting the most effective strategies and components of IoT systems within the interior space. There is a need to have clear guidelines on integrating IoT systems within the interior space to protect security and privacy and provide better healthcare for the aging population. Furthermore, future reviews could dig deeper into the cost-effectiveness of installing and implementing IoT systems within the interior space, either in residential, commercial, or healthcare facilities that will benefit the most.

Limitations of IoT systems within the interior space were discussed, and the most critical parts were based on the security of the collected data and privacy issues of users' data. Also, the usability and technical knowledge needed to operate the technology within space can be a barrier for some users. However, this can be overcome with proper training, doing periodic checks, and visiting the users, and involving a systems evaluation in the post-occupancy evaluation process of the building.

### 5.1 Research grap and limitation

While there has been significant research into the application of IoT systems in interior spaces, several gaps and limitations in research persist that warrant further exploration. Those gaps and limitation are as follows:

Integration of IoT systems: Despite the existence of several standalone IoT systems targeting different areas of a home or commercial space, there is a lack of comprehensive studies that integrate these systems seamlessly for an enhanced user experience. This study addresses this gap by examining how various IoT solutions can work in tandem to optimize overall functionality.

User-friendly IoT systems: Several studies, such as [2], have highlighted that the adoption of IoT systems is influenced by users' perceived IoT abilities and internet proficiency. Therefore, there is a need for research focusing on creating more intuitive, user-friendly IoT systems that cater to all demographic groups, regardless of their technical skills.

Privacy and security of IoT systems: Many researchers, like [1], have pointed out that the current state of IoT systems could present significant privacy and security risks. While some attempts have been made to address these issues, comprehensive solutions are still largely lacking. This paper seeks to highlight these concerns and advocate for research prioritizing robust privacy and security measures in IoT systems.

Standardization of IoT systems: A lack of standardization exists in the current IoT landscape, with various manufacturers using different technologies and standards. This situation often leads to compatibility issues, hindering the adoption of IoT technologies. Therefore, more research on the development of standardized protocols and interfaces is needed.

Energy-efficient IoT systems: While IoT systems have been shown to help manage energy consumption, more research needs to be conducted on making the IoT devices themselves more energy-efficient. This area forms a significant part of this paper's scope.

IoT systems in education: The integration of Internet of Things (IoT) technology in the educational sector holds significant promise, transforming learning environments and teaching methodologies. Smart, interactive classrooms can be created through IoT, where sensors monitor and adapt environmental conditions for optimal learning [34]. IoT devices can facilitate learning analytics, collecting data about students' learning habits and performance, enabling tailored educational strategies [10,33]. Furthermore, interconnected student devices can promote collaborative learning experiences [33].

The application of IoT extends to enhancing e-learning platforms, offering real-time, interactive sessions and immersive experiences through Virtual Reality (VR) and Augmented Reality (AR) devices [13]. It also promotes safety in educational institutions via surveillance systems and emergency alerts and can improve accessibility for students with disabilities [12]. However, the increased use of IoT in education introduces challenges, such as privacy concerns, the need for digital literacy, and issues around equitable access [3]. The vast opportunities and significant challenges presented by the intersection of IoT, and education underline the need for continued research in this field.

As we look into the future, there are sevelate action that can be take to fill the gap in the literature in the field of IoT and interior design. Hence, the need for an in-depth empirical research that focuses on the long term benefits of implementing IoT technology within the interior space. These studidies can give new insights and explore the effectiveness of using such technologies, and what extents it should be used.

Particularly for interior spaces, future research could explore innovative IoT applications that provide comprehensive security solutions. They might investigate how new advancements, such as artificial intelligence and machine learning, can be integrated into IoT systems to enhance their effectiveness.

As for interior design, future research could explore the extent of using IoT technology in improving safety and securitybsuliosions within the built environment. Fithurmore, in the ducation sectore, researchers can explorethe utilization of IoT technology in creating more engaging and interactive classroom space.

In conclusion, there is a huge potential for IoT technology for enhancing interior design and the built environment on a multy level scale. It can evolutionze safety, security, power consumption, and education. However, the existing literature in these specifics areas is lacking the depth and comprehensiveness. This review paper has identified the gaps, and it provides an overview of the current state of research in this field. By addressing these gaps, the field of IoT technology can be future studied which will lead to maximizing it benifitial usage, and create safer and more effieciant interior spaces.

By addressing these research gaps, a profound contribution to the advancement of IoT systems' deployment in interior spaces, emphasizing their potential to enhance safety, efficiency, and overall user experience can be made.

## Ethics declariations

Informed consent was not required for this study because the data used in this paper is published research, and no data collection from human and/or animal subjects were conducted.

## Declaration of competing interest

The authors declare that they have no known competing financial interests or personal relationships that could have appeared to influence the work reported in this paper.
